# Transsaccadic integration benefits are not limited to the saccade target

**DOI:** 10.1152/jn.00420.2019

**Published:** 2019-07-31

**Authors:** Emma E. M. Stewart, Alexander C. Schütz

**Affiliations:** Allgemeine und Biologische Psychologie, Philipps-Universität Marburg, Marburg, Germany

**Keywords:** eye movement, saccade, transsaccadic integration

## Abstract

Across saccades, humans can integrate the low-resolution presaccadic information of an upcoming saccade target with the high-resolution postsaccadic information. There is converging evidence to suggest that transsaccadic integration occurs at the saccade target. However, given divergent evidence on the spatial specificity of related mechanisms such as attention, visual working memory, and remapping, it is unclear whether integration is also possible at locations other than the saccade target. We tested the spatial profile of transsaccadic integration, by testing perceptual performance at six locations around the saccade target and between the saccade target and initial fixation. Results show that integration benefits do not differ between the saccade target and surrounding locations. Transsaccadic integration benefits are not specific to the saccade target and can occur at other locations when they are behaviorally relevant, although there is a trend for worse performance for the location above initial fixation compared with those in the direction of the saccade. This suggests that transsaccadic integration may be a more general mechanism used to reconcile task-relevant pre- and postsaccadic information at attended locations other than the saccade target.

**NEW & NOTEWORTHY** This study shows that integration of pre- and postsaccadic information across saccades is not restricted to the saccade target. We found performance benefits of transsaccadic integration at attended locations other than the saccade target, and these benefits did not differ from those found at the saccade target. This suggests that transsaccadic integration may be a more general mechanism used to reconcile pre- and postsaccadic information at task-relevant locations.

## INTRODUCTION

As humans move their eyes across the visual field, upcoming saccade targets are selected using low-resolution peripheral vision and subsequently brought under higher resolution foveal scrutiny after the saccade ends. Transsaccadic integration is one mechanism that allows us to reconcile the low-resolution presaccadic view of an object at the saccade target with the high-resolution postsaccadic percept of that object, thus contributing to the maintenance of perceptual stability across saccadic eye movements. Transsaccadic integration has primarily been tested at the saccade target; however, given that there is divergent evidence on the spatial profile and specificity of related transsaccadic processes such as remapping, attention, and memory, it is important to determine the extent of integration benefits at locations other than the saccade target.

There is a growing amount of literature (Aagten-Murphy and Bays 2018) to suggest that the visual system can integrate intrinsic stimulus properties such as color (Oostwoud Wijdenes et al. 2015; Wittenberg et al. 2008), form (Demeyer et al. 2010), orientation (Ganmor et al. 2015; Wolf and Schütz 2015), and numerosity (Hübner and Schütz 2017), and that it can do so in a near-optimal manner (Ganmor et al. 2015; Wolf and Schütz 2015). Recent studies have also provided evidence for the fusion of pre- and postsaccadic stimulus properties (Paeye et al. 2017) and have suggested that our postsaccadic percept is influenced by presaccadic stimulus properties (Fabius et al. 2016, 2019). These studies all contravene earlier claims against transsaccadic integration (i.e., O’Regan and Lévy-Schoen 1983; Rayner and Pollatsek 1983) and provide converging evidence that transsaccadic integration is possible and that our ultimate percept of a saccade stimulus depends on both pre- and postsaccadic information. Whereas these studies focused on integration at the saccade target location, a recent study reported broader transsaccadic integration benefits: Schut et al. (2018) showed that integration of pre- and postsaccadic feature information occurred even when saccades landed up to 4° from the saccade target. Furthermore, we showed (Stewart and Schütz 2019) that integration benefits can occur for locations along the saccade trajectory, even when the pre- and postsaccadic stimuli are processed in different hemifields.

Evidence also suggests that it is not only the information that is contained within a stimulus that is integrated but also information about the location of the stimulus itself. Prime et al. (2006) demonstrated that location information could be transferred across saccades and that humans can use extraretinal signals to adjust for changing eye position and incorporate this location information into the transsaccadic percept. Cicchini et al. (2013) also investigated integration of stimulus position across saccades, finding that the locations of objects with similar properties that appear along the saccadic path during the saccade are integrated. This study again suggests that integration may not be confined to purely peripheral to foveal locations in the visual field.

Higher level processes such as attention and visual working memory (VWM) have been implicated as mechanisms underlying transsaccadic integration (Stewart and Schütz 2018a, 2018b), potentially due to the guidance of attentional pointers in the visual field (Cavanagh et al. 2010). There are divergent findings on the spatial specificity of presaccadic attention, with some studies claiming that attention is locked to the saccade target (e.g., Deubel and Schneider 1996; Hoffman and Subramaniam 1995; Kowler et al. 1995) and others showing that attention may also spread to locations around the saccade target (Castet et al. 2006; Harrison et al. 2012; Stewart and Ma-Wyatt 2017). In addition, attention can also be allocated to a location other than the saccade target during a saccade, when the alternate location is task relevant (White et al. 2013; Yao et al. 2016a). In paradigms investigating sequences of saccades, it has been shown that multiple impending saccade locations can be attentionally selected in parallel, providing evidence that multiple task-relevant locations can receive attentional benefits (Baldauf and Deubel 2008; Gersch et al. 2009; Rolfs et al. 2011).

The spatial specificity of VWM during saccades has similarly shown that objects near the saccade target are remembered better than other locations (Irwin 1996; Irwin and Andrews 1996), and change detection is better near the saccade target (Henderson and Hollingworth 1999, 2003). This suggests that VWM may be prioritized at the saccade target (Hanning et al. 2016; Ohl and Rolfs 2017, 2018); however, if participants were instructed to attend to a location other than the saccade target, memory performance was equally high at those attended locations (Irwin and Gordon 1998), indicating that memory resources can be allocated to relevant locations across the visual field. Given that some evidence suggests that the saccade target receives preferential treatment in terms of attention and VWM, and others suggest that these resources can be more widespread, it is unclear whether integration will also be necessarily coupled to the vicinity of the saccade target or whether integration benefits can occur in a broader manner across the visual field.

**Fig. 1.**
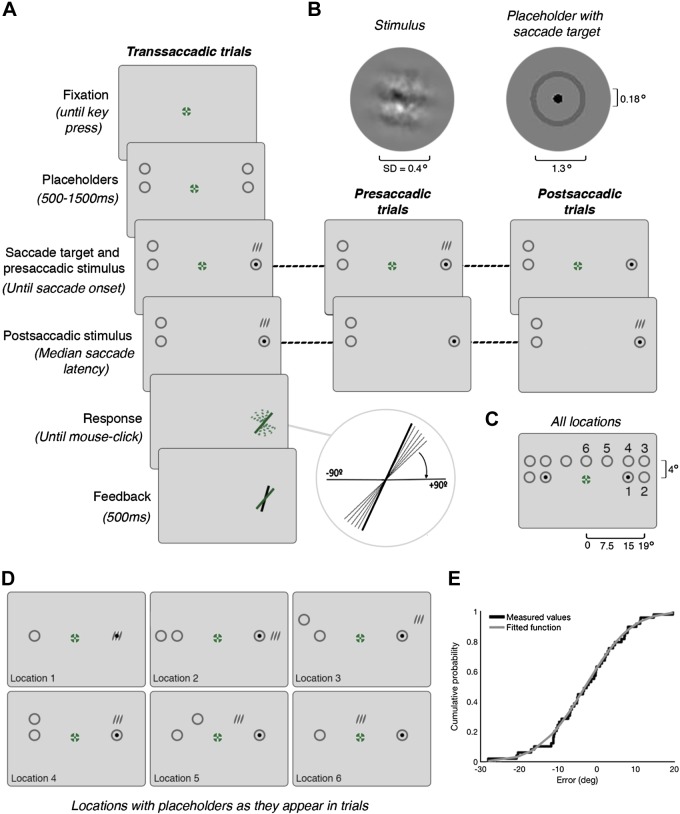
*A*: timeline of events in a trial. In presaccadic trials the perceptual stimulus appeared until saccade onset, in postsaccadic trials the perceptual stimulus appeared after saccade onset, and in transsaccadic trials the perceptual stimulus appeared before and after the saccade. Responses were given by rotating an on-screen bar to match stimulus orientation, and feedback was given by displaying the reported and actual stimulus orientations together. *B*: perceptual stimulus and placeholder with saccade target. *C*: all locations tested. *D*: individual *locations 1–6* with placeholders as they would appear in a given trial for that location. Diagrams are not drawn to scale. *E*: example cumulative distribution fitted to error measurements for one subject and one condition. The just-noticeable difference (JND) is measured as the SD of this curve.

In this study, we aimed to determine the spatial profile of transsaccadic integration at locations other than the saccade target, and unlike previous studies (Cicchini et al. 2013; Prime et al. 2006), we were looking not at the integration of location information itself, but at the integration of stimulus feature information at different, precued locations. We tested integration at six locations: the saccade target, beyond the saccade target, above the saccade target, above and beyond the saccade target, a location between initial fixation and saccade target, and a location above initial fixation (see Fig. 1 and Table 1).

## METHOD

### Equipment

Stimuli were presented using a back-projection setup, using a PROPix projector (VPixx Technologies) with a resolution of 1,920 × 1,080, and a refresh rate of 120 Hz, with a 91-cm × 51-cm screen from Stewart Filmscreen. Viewing distance was 106 cm. Background luminance was 92 cd/m^2^, and the screen was calibrated to ensure a linear gamma correction and to correct the central hot spot. Eye movements were recorded using an EyeLink 1000 (SR Research, Ontario, Canada) with a sampling rate of 1,000 Hz. The experiment was presented with custom-written software in MATLAB using the Psychophysics Toolbox (Brainard 1997; Pelli 1997). Participants responded using a standard keyboard.

### Participants

There were 26 participants (5 men, 21 women), with ages ranging from 18 to 33 yr. All had normal or corrected-to-normal vision and were naive as to the purposes of the experiment. The experiments were carried out in accordance with the Declaration of Helsinki, and ethics approval was obtained from the local ethics commission of the Department of Psychology of Marburg University (proposal number 2015-35k).

### Experimental Procedure

#### Stimuli.

The initial fixation target was a combination of bulls eye and cross hair in shape (Thaler et al. 2013). This was presented in a random color on each trial, generated in DKL color space (Derrington et al. 1984) with a set Cartesian value of 0.4 in the L+M axis, 0.6 on the L-M axis, and 0 on the S axis. The polarity of these values was randomized across trials to avoid the buildup of afterimages. Placeholders were dark gray rings with a diameter of 1.3° and a luminance of 4.3 cd/m^2^. The saccade stimulus was a small black dot with a diameter of 0.18° and a luminance of 2.1 cd/m^2^ (Fig. 1*B*). Perceptual stimuli were oriented Gabors, presented at a random orientation (from 0° to 180°) on each trial. The Gabors had a standard deviation of 1° and a spatial frequency of 2c/°, and were overlaid with bandpass-filtered noise with a central frequency of 2c/° and a Gaussian standard deviation of 1°, and a Gaussian window with a standard deviation of 0.4°. To equate perceptual performance at the different locations before and after the saccade, stimuli were presented at contrast values determined in a pilot experiment. In that pilot experiment, pre- and postsaccadic contrasts at the saccade target were measured separately using a QUEST paradigm set to 82% threshold: the experimental procedure was identical to the main experiment. Based on the contrast threshold measurements from the pilot experiment, stimuli at the saccade target were presented at a contrast of 24% for presaccadic stimuli and 21% for postsaccadic stimuli. This value was then multiplied by a factor for each location, determined by obtaining postsaccadic threshold measurements for each location from seven participants (one participant also completed the main experiment). For the six locations (Fig. 1), baseline contrast values were multiplied by 1.00, 1.00, 1.74, 1.35, 1.20, and 1.38.

#### Procedure.

We tested performance on three eye-movement conditions at six locations (Table 1). Stimulus locations were blocked, and participants were informed where the stimulus would appear before the start of the block. Before the start of the experiment, participants completed a practice block to familiarize themselves with the task and locations.

**Table 1.** Horizontal and vertical coordinates of stimulus locations relative to the screen center and resulting saccade latencies and horizontal amplitudes

Stimulus Location and Condition	Horizontal Eccentricity	Vertical Eccentricity	Saccade Latency, ms	Saccade Amplitude, deg
*Location 1* (saccade target)				
All conditions	15	0	218 (51)	14.5 (0.84)
Presaccadic			218 (48)	14.5 (0.76)
Postsaccadic			229 (57)	14.6 (0.84)
Transsaccadic			219 (46)	14.6 (0.79)
*Location 2*				
All conditions	19	0	237 (57)	15.3 (0.91)
Presaccadic			234 (59)	15.3 (0.95)
Postsaccadic			246 (58)	14.6 (0.91)
Transsaccadic			232 (55)	15.2 (1.02)
*Location 3*				
All conditions	19	4	233 (57)	14.6 (0.88)
Presaccadic			233 (56)	14.6 (1.02)
Postsaccadic			237 (59)	14.5 (0.88)
Transsaccadic			232 (55)	14.5 (1.04)
*Location 4*				
All conditions	15	4	235 (55)	14.3 (0.87)
Presaccadic			228 (53)	14.3 (0.83)
Postsaccadic			236 (56)	14.5 (0.87)
Transsaccadic			232 (55)	14.3 (0.88)
*Location 5*				
All conditions	7.5	4	240 (59)	14.6 (0.90)
Presaccadic			244 (57)	14.6 (0.92)
Postsaccadic			238 (57)	14.5 (0.90)
Transsaccadic			245 (65)	14.5 (0.97)
*Location 6* (above initial fixation)				
All conditions	0	4	246 (60)	14.8 (0.86)
Presaccadic			252 (56)	14.8 (0.85)
Postsaccadic			243 (64)	14.6 (0.86)
Transsaccadic			250 (59)	14.8 (0.89)

Values are medians (interquartile range, IQR) for saccade latency and means (SD) for saccade amplitude.

#### Presaccadic trials.

Participants fixated the central fixation cross and pressed the space bar to begin. Placeholder stimuli appeared at the saccade target and the tested location (Fig. 1*D*) for a random delay between 500 and 1,500 ms. After this time, a saccade target appeared in the center of the saccade target placeholder, on either the left or right (Fig. 1*B*). The perceptual target appeared at the tested location for that block, either replacing the placeholder at the saccade target (*location 1*) or at the other placeholder location shown (*locations 2–6*). In presaccadic trials, the perceptual stimulus (oriented Gabor) appeared at the same time as the saccade target and disappeared when a saccade was detected. Saccade onset was measured as the time when the eye position had moved 2° from the central fixation in a horizontal direction: the stimulus switch occurred on the next frame after this criterion was met. After the saccade, participants used a mouse to rotate an on-screen bar to match the perceived orientation of the stimulus and clicked to confirm. Feedback was given by displaying the selected stimulus orientation together with a black bar showing the actual stimulus orientation.

#### Postsaccadic trials.

Postsaccadic trials followed the same procedure as presaccadic trials, except that the saccade target (black dot) was presented until saccade onset, at which time the perceptual stimulus appeared. This stimulus was then presented for the median saccade latency measured across the previous 20 trials, after which the response was given.

#### Transsaccadic trials.

In transsaccadic trials, the procedure was again identical to presaccadic trials, except that the perceptual stimulus was presented both presaccadically (as in presaccadic trials) and postsaccadically (as in postsaccadic trials). The pre- and postsaccadic stimulus orientation was identical in these trials; contrast was adjusted to account for differences in pre- and postsaccadic stimulus visibility. As in the other conditions, the presaccadic stimulus was presented for the saccade latency on that trial; the postsaccadic stimulus was presented for the median saccade latency across the previous 20 trials.

#### Analyses.

Perceptual performance was measured as the smallest possible angle between shown and reported orientation. A cumulative Gaussian function was fitted to the distribution of signed errors for each condition, and the just-noticeable difference (JND) was calculated as the standard deviation of this fitted distribution. To account for any differences in overall performance between locations, we normalized performance by the mean JND across all conditions (pre-, post-, and transsaccadic) at each location.

#### Predicted performance.

To determine whether optimal integration occurred, we used maximum likelihood estimation (MLE) to calculate the predicted integration performance, based on the reliabilities of the pre- and postsaccadic performance alone. Individual reliabilities for pre- and postsaccadic performance were calculated as

(1) $$rel=\frac{1}{JND^{2}}\text{.}$$

Predicted transsaccadic reliability is calculated as the sum of the pre- and postsaccadic reliabilities (Ernst and Bülthoff 2004):

(2) $$rel_{int}=rel_{per}+rel_{fov}\text{.}$$

The JND for this predicted performance is then calculated as

(3) $$JND_{int}=\sqrt{\frac{1}{rel_{int}}}\text{.}$$

We quantify the relative difference between best single and transsaccadic performance as the difference between best single performance (pre- or postsaccadic) and observed transsaccadic performance, divided by transsaccadic performance:

(4) $$Relative\;difference=\frac{JND_{best\;single\;}-JND_{trans\left(obs\right)}}{JND_{trans\left(obs\right)}}\text{.}$$

#### Exclusions.

Trials were excluded where the saccade latency was below 50 ms or above 2 SD from the median latency. Trials were also excluded when the final eye position was more than 2° above or beyond the saccade target, had an amplitude of less than 10 degrees, and additionally, more than 2SD radial error from the mean landing position. This aimed to exclude trials where participants made eye movements to the surrounding locations. Additionally, trials in which extreme saccade curvature was measured were excluded, where quadratic curvature exceeded ±50. These exclusions led to 23% of trials being excluded across all participants, with 1.3% of trials excluded due to technical eye-tracker error. This resulted in a total of 20,127 trials of 26,208 total recorded trials being included across all participants.

Participants were further excluded when their mean performance across all conditions was more than 2 SD from mean performance across all participants: two participants were excluded on this basis. For each remaining participant, we wanted to ensure that pre- and postsaccadic performance was equated at each location: according to the MLE model, integration benefits will be greatest when pre- and postsaccadic performance is matched and will decrease as performance on the individual conditions becomes further apart. Thus we wanted to ensure that we could observe maximal potential integration benefits and that this potential for integration was equated across locations. We aimed to equate performance by presenting pre- and postsaccadic stimuli at differing contrast levels determined in a pilot study; however, there was still some inequality between pre- and postsaccadic performance for some subjects at some locations. To ensure that performance was equated across all participants and locations, we calculated the ratio of the difference between best single performance (pre- or postsaccadic) and predicted performance, versus the difference between worst single performance and predicted performance pre- to postsaccadic performance, for each location and participant. This ratio ranges from 0, when the predicted performance is equal to the best single performance, to 1, when single performances are equal and predicted integration benefits are maximal. On this basis, we excluded data from participants at a location if the ratio of pre- and postsaccadic performance was <0.1 (per Stewart and Schütz 2018b). These exclusion criteria were not based on actual benefit to integration, but rather potential integration benefits that could be measured given the disparity between pre- and postsaccadic performance. This resulted in data from 35 of 144 locations across all participants being excluded.

#### Mixed model and Bayes factor analyses.

All mixed models were calculated using the nlme package in R (Pinheiro et al. 2019). Fixed effects of location and condition were categorically coded. Random effects were structured as random slopes and intercepts for each participant.

Bayes factors for mixed models were calculated using the BayesFactor package in R (Morey and Rouder 2013), using default priors (inverse gamma prior on the regression and Jeffreys prior on effects). Bayes factors for main effects were calculated as the ratio of evidence for the model containing only that factor vs. the null model (intercept and random effects only). Interactions were calculated as the model containing main effects with no interaction term vs. the full model. Bayes factors for ANOVAs used a g-prior of variance and Jeffreys prior on effects.

## RESULTS

### Best Single Performance vs. Transsaccadic Performance

**Fig. 2.**
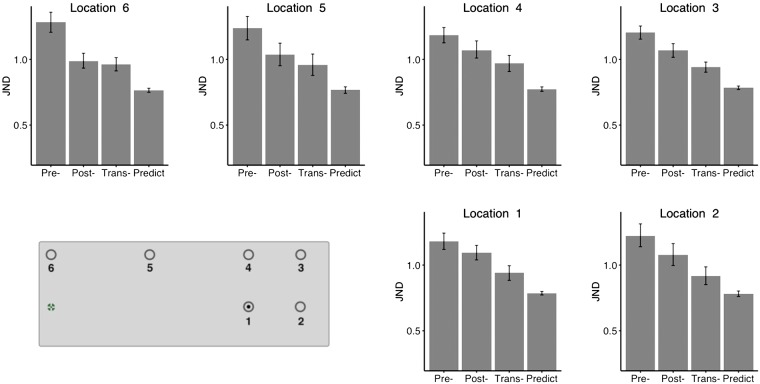
Performance at each location for presaccadic (Pre-), postsaccadic (Post-), transsaccadic (Trans-), and predicted (Predict) optimal integration performance. Values are mean just-noticeable difference (JND). Error bars are 95% confidence intervals.

**Fig. 3.**
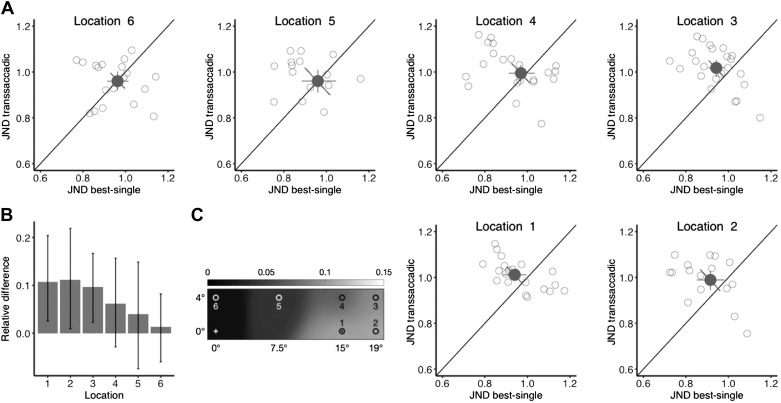
*A*: best single performance (pre- or postsaccadic) vs. transsaccadic performance for each location. Open circles represent individual participants; closed circles are the mean across participants. Values are mean just-noticeable difference (JND). Error bars are 95% confidence intervals (CI). Diagonal error bars represent the error of the difference between best single performance and transsaccadic performance. *B*: integration benefit at each location. Error bars are 95% CI. *C*: heatmap of mean integration benefit relative to observed transsaccadic performance. This heatmap was determined using the mean integration benefit value at each location: values at areas in between the tested locations were interpolated on the basis of weighted values of the nearest tested locations and the distance between these points. As such, these interpolated values were more reliant on the nearest tested location, and this dependence decreased with distance. Note that values for the area below *locations 5* and *6* are based purely on the data from these locations in the absence of data along the saccade path.

To determine whether integration performance differed at the six tested locations (Fig. 2), we compared transsaccadic performance with best single performance (pre- or postsaccadic, at each location; Fig. 3*A*). For integration benefits to occur, transsaccadic performance should be better than best single performance. We ran a linear mixed model with fixed effects of location (*locations 1–6*) and eye-movement condition (transsaccadic or best single) with a random effect of participant. There was a significant effect of eye-movement condition [*F*(1,119) = 7.81, *P* = 0.0061], but there was no significant effect of location [*F*(5,95) = 0.44, *P* = 0.82] and no interaction between location and eye-movement condition [*F*(5,119) = 0.99, *P* = 0.43]. The estimate and 95% confidence interval (CI) for difference in eye-movement condition across all locations are −0.036, 95% CI [−0.071, −0.00027]. Estimates and 95% CI for difference between eye-movement conditions at all locations are as follows: *location 1*, −0.07 [−0.14, −0.00055]; *location 2*, −0.074 [−0.15, 0.0063]; *location 3*, −0.075 [−0.14, −0.0075]; *location 4*, −0.026 [−0.092, 0.041]; *location 5*, −0.00097 [−0.081, 0.079]; and *location 6*, 0.0028 [−0.071, 0.077]. This suggests that transsaccadic performance was better than best single performance, and this did not differ significantly across locations. To provide further evidence that there is a null effect of location, we performed a Bayesian mixed model with the same fixed and random effects as the frequentist model. There was moderate evidence for a main effect of eye-movement condition (BF_10_ = 6.26) and strong evidence in favor of a null effect of location (BF_10_ = 0.02), but no evidence in favor of an interaction between eye-condition and location (BF_10_ = 0.13). Nevertheless, at *location 6*, above the initial fixation location, the best single performance and the transsaccadic performance were on average very similar, whereas the transsaccadic performance was on average better than the best single performance at all other locations.

We quantified the relative difference between best single performance and transsaccadic performance as a ratio of best single performance (*Eq. 4*). To determine whether this relative difference differed between locations, we used a mixed model with fixed effect of location and random effect of participant. There was no significant effect of location [*F*(5,95) = 0.66, *P* = 0.65, BF_10_ = 0.06]. Estimates and 95% CI for each location in comparison with *location 1* (baseline) are as follows: *location 2*, −0.0048 [−0.22, 0.21]; *location 3*, 0.0098 [−0.18, 0.20]; *location 4*, 0.045 [−0.15, 0.28]; *location 5*, 0.066 [−0.14, 0.28]; and *location 6*, 0.093 [−0.11, 0.29]. To better visualize the spread of integration benefits across locations, Fig. 3*C* shows integration benefit as a heatmap.

### Transsaccadic vs. Predicted Performance

To determine whether integration performance was optimal according to the MLE model, we compared observed transsaccadic performance with predicted transsaccadic performance (Fig. 2). We ran a mixed model with fixed effects of condition (observed or predicted performance) and location, and random effect of participant. There was a significant effect of eye-movement condition [*F*(1,119) = 171.33, *P* < 0.0001, BF_10_ = 6.3e27], but no significant effect of location [*F*(5,95) = 0.20, *P* = 0.96, BF_10_ = 0.009] and no interaction between eye-movement or location [*F*(5,119) = 0.65, *P* = 0.66, BF_10_ = 0.072]. The estimate for difference in eye-movement condition across all locations was 0.077, 95% CI [0.05, 0.11]. Estimates and 95% CI for difference between eye-movement conditions at all locations are as follows: *location 1*, 0.15 [0.092, 0.22]; *location 2*, 0.13 [0.06, 0.20]; *location 3*, 0.16 [0.099, 0.22]; *location 4*, 0.20 [0.14, 0.25]; *location 5*, 0.19 [0.13, 0.26]; and *location 6*, 0.20 [0.13, 0.26]. This indicates that integration performance, although better than the best single performance, was not optimal, and this was the same at every location. This is surprising given previous work showing near-optimal integration of orientation information (Ganmor et al. 2015; Wolf and Schütz 2015); however, this could be due to split attentional resources across the saccade target and other locations (see discussion for further explanation).

### Saccade Metrics

We compared saccade latency, amplitude, and curvature across locations and conditions to ensure that any effects were not due to differences in saccades across conditions and to measure potential inhibition of, or competition between, potential saccade targets (Buonocore and McIntosh 2008; Walker et al. 1997).

#### Saccade latency.

**Fig. 4.**
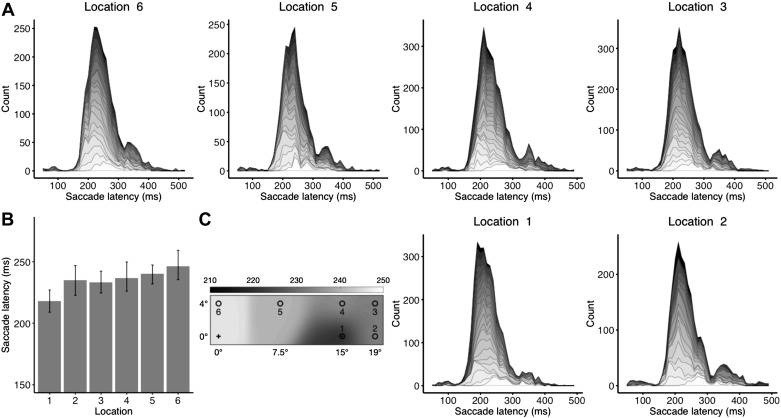
Saccade latency. *A*: distribution of saccade latency for each participant at each location. *B*: mean saccade latency for each location. Error bars are 95% confidence intervals. *C*: heatmap of latency at all locations.

Saccade latency was measured using the EyeLink saccade detection algorithm. The median saccade latency across all participants and locations (including excluded locations) was 234 ms, with an interquartile range of 58 ms. Latencies for individual locations are shown in Table 1 and Fig. 4. A one-way ANOVA revealed a significant effect of location on saccade latency [*F*(5,126) = 2.99, *P* = 0.014; BF_10_ = 2.68], with post hoc multiple comparisons with a Holm correction revealing a significant difference only between *location 1* (saccade target) and *location 6* [*t*(126) = −3.61, *P* = 0.0066]. Because *location 6* is above initial fixation, saccades in this condition would be being made away from the perceptual target. There was no significant effect of eye-movement condition (pre-, post-, or transsaccadic) on saccade latency [*F*(2,75) = 0.23, *P* = 0.80; BF_10_ = 0.13]. This demonstrates that saccade latency did not affect performance in individual conditions.

#### Saccade amplitude.

Mean horizontal saccade amplitude across all participants and locations was 14.6° (SD 0.99). Saccade amplitudes for individual locations are shown in Table 1. A one-way ANOVA revealed a significant main effect of location on saccade amplitude [*F*(5,126) = 11.48, *P* < 0.0001]. Post hoc multiple comparisons with a Holm correction show a significant difference between *locations 1* and *2* [*t*(126) = −5.61, *P* = <0.0001], *locations 2* and *4* [*t*(126) = −6.65, *P* = <0.0001], *locations 3* and *2* [*t*(126) = −5.0, *P* < 0.0001], and *locations 2* and *5* [*t*(126) = 5.16, *P* < 0.0001]. All other locations did not show a significant difference. In *location 2*, the perceptual target was beyond the saccade target, which led to an increase of saccade amplitudes. However, the differences in amplitude were within about a half degree, quite small compared with the actual distance of the perceptual target of 4°. There was also no significant difference between eye-movement conditions (pre-, post- or transsaccadic) on saccade amplitude [*F*(2,75) = 1.13, *P* = 0.33; BF_10_ = 0.27].

#### Saccade curvature.

**Fig. 5.**
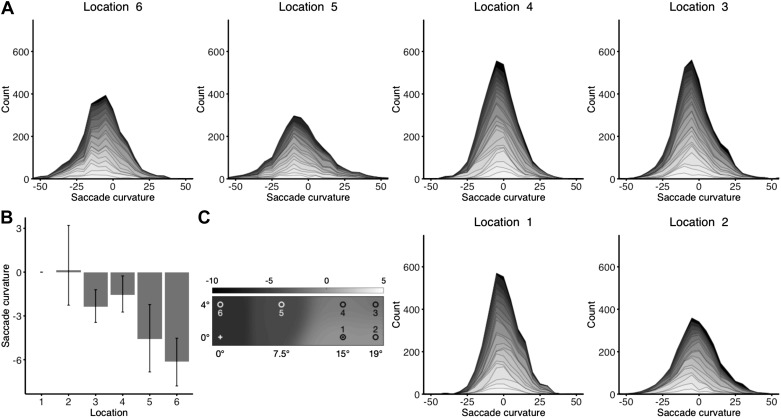
Saccade curvature. *A*: distribution of saccade curvature across all participants for each location. *B*: mean saccade curvature for each location compared with baseline curvature at *location 1*. Error bars are 95% confidence intervals. *C*: heatmap of curvature at all locations.

We calculated saccade curvature for each stimulus location, to investigate any potential effects of attentional suppression arising from competing potential saccade targets (Sheliga et al. 1994). To quantify saccade curvature, we fitted a quadratic function to each saccade trace: the curvature was measured as the quadratic coefficient of the second-order fit (Ludwig and Gilchrist 2002). Figure 5*A* shows distributions of saccade curvature for all locations, and Fig. 5*B* shows average saccade curvature as a difference from baseline curvature at *location 1* (saccade target). Positive values represent curvature toward the top of the screen (toward stimulus locations), and negative values represent curvature toward the bottom of the screen (away from stimulus locations). A mixed model revealed a significant effect of location [*F*(5,94) = 13.79, *P* < 0.0001]. Estimates and 95% CI for each location in comparison with *location 1* (baseline) are as follows: *location 2*, 0.19 [−2.1, 2.48]; *location 3*, −2.51 [−4.63, −0.39]; *location 4*, −1.50 [−3.62, 0.62]; *location 5*, −6.05 [−8.23, −3.87]; and *location 6*, −6.88 [−9.13, −4.63]. Post hoc pairwise comparisons with a Holm correction showed a significant difference between *location 1* and *location 5* [*t*(94) = 5.51, *P* = <0.0001], with an estimate of 6.05 [2.7, 9.4], and between *location 1* and *location 6* [*t*(94) = 6.07, *P* < 0.0001], with an estimate of 6.88 [3.5, 10.3]. This curvature away from the nontarget stimuli could represent an attentional inhibition of these locations (Sheliga et al. 1994, 1995), and this is especially evident for locations close to initial fixation (Van der Stigchel and Theeuwes 2005).

## DISCUSSION

The results of this study suggest that transsaccadic integration, i.e., an increase in transsaccadic performance compared with pre- and postsaccadic performance, can occur at locations other than the saccade target when the integration location is behaviorally relevant, and that integration benefits at locations surrounding the saccade target do not differ from those at the saccade target itself. Pre- and postsaccadic performances were equated and stimulus locations were cued to measure transsaccadic integration per se, and to limit the influence of other relevant factors such as differences in performance across the visual field, or presaccadic attention shifts. Although performance was not optimal based on predictions from pre- and postsaccadic performance alone, the benefits from transsaccadic performance compared with best single performance did not differ between locations. This is consistent with studies showing that integration can occur beyond the saccade end point (Schut et al. 2018) and with studies showing that integration of location information can occur across the visual field across saccades; for example, Prime et al. (2006) demonstrated that integration occurs beyond the fovea, in the absence of any common visual cues between pre- and postsaccadic vision, suggesting that integration may occur in a broader manner across the visual field and may rely on internal oculomotor signals to align the pre- and postsaccadic stimuli. Cicchini et al. (2013) also saw that integration could occur at locations other than the saccade target, and the properties of perisaccadic object shifts were consistent with the spatiotemporal properties of remapped receptive fields. It must be noted, however, that the integration benefits observed in this study were measured under ideal conditions, where pre- and postsaccadic performance were equated at each location in order to observe maximum potential benefits. We also accounted for differences in visual sensitivity across the visual field and aimed to equate absolute performance levels at all locations. This demonstrates that whereas it is possible to observe integration benefits at nontarget locations, actual benefits may differ in a real-world scenario where pre- and postsaccadic reliability is not matched, and might decline with eccentricity. It should also be noted that although there was no significant difference in integration between locations, there is a trend toward a smaller integration benefit as distance from the target increases: it may be the case that this effect exists but is too subtle to be measured by this paradigm.

### Attention and Memory

The spatial pattern of integration benefits could also be reflective of attentional allocation to these task-relevant locations across the saccade. Whereas many studies have shown a specificity of presaccadic attention at the saccade target (Deubel and Schneider 1996; Hoffman and Subramaniam 1995; Kowler et al. 1995), there is also evidence to suggest that attentional benefits may be observed at surrounding locations (Castet et al. 2006; Harrison et al. 2012; Stewart and Ma-Wyatt 2017; Stewart et al. 2019), and that locations other than the saccade target can be attentionally selected based on both features, and the location of behaviorally relevant stimuli on previous trials (White et al. 2013). This attention to task-relevant, nonsaccade targets emerges as rapidly as 30 ms after a saccade (Yao et al. 2016a). Our results suggest that transsaccadic integration benefits can occur at locations other than the saccade target, which implies that some level of attentional resource must also be available at those nontarget locations and may also be reflective of a link between attention and remapping across saccades (Szinte et al. 2016, 2018). Whereas integration performance did not differ significantly across locations, there was a slight (but nonsignificant) difference in performance at *location 6*. This location was situated above the initial fixation point, so in making a saccade to the saccade target, participants were saccading away from the perceptual target. This could reflect patterns of suppression at perceptual targets during anti-saccade tasks (Mikula et al. 2018), and the greater saccade curvature at *locations 5* and *6* also seems to reflect an attentional inhibition of these locations (Sheliga et al. 1994). Seeing more prominent integration effects in the direction of the saccade also reflects the zone in which remote distractor effects are most pronounced (Walker et al. 1997). These indicators of attentional inhibition may be reflective of a planned, but canceled, saccade to the other stimulus locations; however, even if this is the case, it does not negate the finding that integration can occur at multiple attended targets in disparate locations in the visual field.

This study does not disambiguate whether this is an automatic shift of attention or memory to all potential locations, or whether locations are attentionally selected on the basis of their relevance for impending integration (Melcher 2009); however, it seems likely that locations were selected on the basis of task relevance, thereby providing attentional pointers for subsequent integration to occur (Cavanagh et al. 2010; Mathôt and Theeuwes 2011).

Similarly, although studies have suggested that the saccade target receives prioritized access to VWM resources (Ohl and Rolfs 2017, 2018) and that transsaccadic memory performance should be better at the saccade target (Henderson and Hollingworth 1999, 2003; Irwin 1996; Irwin and Andrews 1996), we saw no evidence of larger integration benefits at the saccade target than at any other location. This is in line with Irwin and Gordon (1998), who showed transsaccadic memory benefits at locations other than the saccade target, when that other location was behaviorally relevant, and suggests again that if a location is flagged as relevant, then it can receive attention and memory resources and benefit from transsaccadic integration. As such, one factor that may have influenced these results is the predictability of the tested locations. Locations were blocked to avoid any effects of uncertainty: if the tested location was unpredictable, then there would have been an inherent imbalance between single trials and transsaccadic trials, because pre- and postsaccadic stimuli would not be predictable in single trials, but the postsaccadic stimulus would always be predictable in transsaccadic trials. Performance would also become reliant mainly on exogenous attentional capture: we wanted to negate any effects of attentional capture by making the locations predictable so that any effects could be attributed to integration alone. This does however raise the question of whether integration performance would have been different had the locations not been predictable and flagged as task relevant. This also raises a related issue, that the placeholder stimuli were switched off before the pre- or postsaccadic stimulus was presented: this could have drawn additional attention to that location, which may have influenced the processing of the Gabor presented immediately after the disappearance of the placeholder. However, it is likely that this would have affected pre- or postsaccadic performance rather than integration itself.

### Remapping: Space and Features

Although this study did not aim to directly test any link between patterns of receptive field shifts and transsaccadic integration, remapping has been posited as a process that potentially underpins integration. There are two factors to consider when comparing our results with those of the remapping literature: the spatial profile of remapping and the features themselves that may be remapped. There is divergent evidence on the spatial profile of remapping: remapping in the lateral intraparietal area (LIP) can occur across the whole visual field and is not specific to the location of an impending eye movement (Mirpour and Bisley 2012), whereas receptive fields from frontal eye field neurons converge toward the saccade target (Zirnsak and Moore 2014; Zirnsak et al. 2014), in contrast to the shift parallel to the saccade vector in forward remapping. The pattern of broader forward remapping has also been shown to occur for features at locations other than the saccade target (He et al. 2018), supporting the idea that both feature and location information can be updated in a more general manner across the visual field. Indeed, there is ample behavioral evidence that features can be remapped (Harrison et al. 2013; He et al. 2017; Melcher 2007, 2008; Wolfe and Whitney 2015). Although some physiological studies have suggested that features cannot be remapped (Neupane et al. 2016; Rolfs and Szinte 2016; Yao et al. 2016b), evidence for remapping of shape information has been found in LIP (Subramanian and Colby 2014) and transfer of visual feature information in V3, V4 and V0 (Zimmermann et al. 2016). Our study has shown a broad pattern of feature integration at all attended locations, which would be consistent with the broader forward remapping seen in LIP (Mirpour and Bisley 2012), and also the remapping of feature information that is also observed in this area (Subramanian and Colby 2014). Indeed, LIP seems to be a natural potential candidate for integration to occur, because it could combine processes such as remapping and the guidance of attentional pointers to areas of priority in the visual field (Cavanagh et al. 2010).

### Optimality

Previous studies have shown that pre- and postsaccadic orientation information is integrated nearly optimally (Ganmor et al. 2015; Wolf and Schütz 2015); however, this was not the case for any tested location in this study. There are a number of possible, not mutually exclusive explanations for this: *1*) Planning a saccade to one location while performing a perceptual task at another location may have resulted in a division of attentional resources and therefore reduction of integration (Stewart and Schütz 2018a). However, this explanation cannot account for the finding that performance was also not optimal at the saccade target, where there would be no division of resources. *2*) We measured perceptual judgments of orientation using a free-rotation task, which may have incorporated some amount of motor noise, which was then incorporated into the predictions but actually cannot be reduced by transsaccadic integration. *3*) The observed transsaccadic benefits might not originate by integrating independent signals, but merely by the prolonged exposure time of the stimuli in the transsaccadic condition compared with the pre- or postsaccadic conditions. However, we directly tested this in another experiment (Stewart and Schütz 2019) and found that the predicted benefits from prolonged stimulus exposure were in fact greater than those predicted by the MLE model: this explanation would then be counter to the less than optimal transsaccadic benefits observed in the current study. *4*) Unlike some previous studies (Wolf and Schütz 2015), we did not present uninformative placeholders after the saccade in presaccadic conditions and before the saccade in postsaccadic conditions. These placeholders might mask the perceptual stimuli and, as a result, degrade the pre- and postsaccadic performance, which also leads lower to predictions for transsaccadic performance. Since the perceptual stimuli might mask each other in the transsaccadic condition, as well, the predictions based on pre- and postsaccadic performance without masking might overestimate transsaccadic performance. However, optimal transsaccadic performance was observed in some studies even without the use of placeholders (Ganmor et al. 2015; Hübner and Schütz 2017). *5*) Causal inference models (Atsma et al. 2016; Körding et al. 2007; Shams and Beierholm 2010) predict that information is only integrated if it is attributed to the same source. Changing the contrast of the pre- and postsaccadic stimulus might have led to a situation where both stimuli are no longer considered to be the same object and therefore information is not integrated to the full extent.

### Integration as a Mechanism for Perceptual Stability

How is transsaccadic integration a useful mechanism for the maintenance of perceptual stability? Previous studies have shown that integration is reliant on both VWM and attention (Stewart and Schütz 2018a, 2018b), and it is likely that integration can occur at locations that are task relevant or attended, but may not be an automatic mechanism that occurs in a “blanket” fashion across the whole visual field (see Stewart and Schütz 2018b for further discussion). This view is consistent with the current results, given that locations were blocked, so we always tested integration at attended, task-relevant locations. Although the actual observed benefit to integration is quite small, we do not think that the absolute magnitude of the benefit from integration is a relevant quantity to judge the usefulness of integration. Given the assumption of independence of pre- and postsaccadic signals, the maximum possible benefit from integration is statistically limited by MLE: this method has been applied to multisensory integration (Ernst and Bülthoff 2004), where small benefits are also observed. Presaccadic attentional benefits have also been measured in a similarly small range (Li et al. 2016; Rolfs and Carrasco 2012; Stewart et al. 2019). Rather than integration benefits being a definitive measure of how much perceptual stability can be attributed to transaccadic integration, we think rather that these measured benefits are a hint of what might be going on in the visual system: presaccadic information and postsaccadic information are initially processed independently, and if they are attributed to the same source, then they are weighted and integrated into a single transsaccadic percept.

### Conclusion

This study showed that transsaccadic integration benefits can occur at attended, task-relevant locations other than the saccade target, and this integration benefit did not differ from that observed at the saccade target. This suggests that in terms of integration, the saccade target may not receive preferential processing and that integration may rather be a mechanism that reconciles pre- and postsaccadic information across the whole visual field.

## GRANTS

This project has received funding from the European Research Council under the European Union’s Horizon 2020 research and innovation program (Grant Agreement No. 676786).

## DISCLOSURES

No conflicts of interest, financial or otherwise, are declared by the authors.

## AUTHOR CONTRIBUTIONS

E.E.M.S. and A.C.S. conceived and designed research; E.E.M.S. performed experiments; E.E.M.S. analyzed data; E.E.M.S. and A.C.S. interpreted results of experiments; E.E.M.S. prepared figures; E.E.M.S. drafted manuscript; E.E.M.S. and A.C.S. edited and revised manuscript; E.E.M.S. and A.C.S. approved final version of manuscript.

## ENDNOTE

At the request of the authors, readers are herein alerted to the fact that additional materials related to this manuscript may be found at https://doi.org/10.5281/zenodo.3356073. These materials are not a part of this manuscript, and have not undergone peer review by the American Physiological Society (APS). APS and the journal editors take no responsibility for these materials, for the website address, or for any links to or from it.
